# Predicting functional outcome in acute ischemic stroke patients after endovascular treatment by machine learning

**DOI:** 10.1515/tnsci-2022-0324

**Published:** 2023-11-27

**Authors:** Zhenxing Liu, Renwei Zhang, Keni Ouyang, Botong Hou, Qi Cai, Yu Xie, Yumin Liu

**Affiliations:** Department of Neurology, Zhongnan Hospital of Wuhan University, 169 Donghu Road, Wuchang District, 430071, Wuhan, Hubei, China; Department of Neurology, Yiling Hospital of Yichang City, 443100, Yichang, Hubei, China; Department of Neurology, Wuhan Fourth Hospital, 430033, Wuhan, Hubei, China

**Keywords:** nomogram, mechanical thrombectomy, machine learning algorithm, predictive models

## Abstract

**Background:**

Endovascular therapy (EVT) was the standard treatment for acute ischemic stroke with large vessel occlusion. Prognosis after EVT is always a major concern. Here, we aimed to explore a predictive model for patients after EVT.

**Method:**

A total of 156 patients were retrospectively enrolled. The primary outcome was functional dependence (defined as a 90-day modified Rankin Scale score ≤ 2). Least absolute shrinkage and selection operator and univariate logistic regression were used to select predictive factors. Various machine learning algorithms, including multivariate logistic regression, linear discriminant analysis, support vector machine, *k*-nearest neighbors, and decision tree algorithms, were applied to construct prognostic models.

**Result:**

Six predictive factors were selected, namely, age, baseline National Institute of Health Stroke Scale (NIHSS) score, Alberta Stroke Program Early CT (ASPECT) score, modified thrombolysis in cerebral infarction score, symptomatic intracerebral hemorrhage (sICH), and complications (pulmonary infection, gastrointestinal bleeding, and cardiovascular events). Based on these variables, various models were constructed and showed good discrimination. Finally, a nomogram was constructed by multivariate logistic regression and showed a good performance.

**Conclusion:**

Our nomogram, which was composed of age, baseline NIHSS score, ASPECT score, recanalization status, sICH, and complications, showed a very good performance in predicting outcome after EVT.

## 1 Introduction

Ischemic stroke is a major disease threatening human life and health, with high disability and mortality [1]. Previous studies have demonstrated that endovascular thrombectomy (EVT) improved functional outcome for acute ischemic stroke patients due to large vessel occlusion (LVO) [2,3,4,5,6,7]. Therefore, intravenous thrombolysis (IVT) plus EVT, or direct EVT when IVT is contraindicated, are recommended within 6 h of stroke onset. Studies also demonstrated the benefit of EVT in imaging-selected stroke patients due to LVO in the anterior circulation between 6 and 24 h [8,9].

The prognosis of stroke patients due to LVO after EVT has been a concern of clinicians. A meta-analysis showed that 46% of patients after EVT obtained functional independence at 90 days [7]. Meanwhile, two major clinical trials (DIRECT-MT investigation, MR CLEAN–NO IV investigation) have shown that the proportion of good functional outcome after EVT was lower in east Asian patients compared with westerners (functional independence of 36.6 vs 50.1%) [10,11], this could be caused by the higher frequency of intracranial atherosclerotic lesions. The fact that a large proportion of patients treated with EVT could not avoid death or major disability is frustrating. Several studies have been conducted to analyze the clinical prognosis after EVT, and the prognostic factors included age, baseline National Institute of Health Stroke Scale (NIHSS) score, Alberta stroke program early CT (ASPECT) score, etc. [12,13,14,15,16,17]. However, most of the findings were limited to moderate predictive power. Moreover, logistic regression analysis was applied by most of the studies for screening clinical and imaging variables, few other machine learning methods were used to investigate this issue.

In recent years, various machine learning algorithms have been applied in clinical researches, such as linear discriminant analysis (LDA), support vector machine (SVM), *k*-nearest neighbors (KNN), and decision tree algorithms [18,19,20,21]. Furthermore, visualization and scoring systems could help clinicians make clinical decisions easier and faster. Nomogram is a graphical statistical tool which combines variables to generate a continuous scoring system and calculate the precise risk probability of a clinical event for an individual patient [22]. It can serve as an important tool in modern medical decision-making and has been used in medical specialties like cancer and surgery [22,23,24].

The main objective of the present study was to develop and validate various machine learning models to predict 3-month functional outcome after EVT for acute ischemic stroke due to LVO in the anterior circulation. Our aim was to establish a reliable model for predicting functional outcome in stroke patients after EVT, thus guiding clinical management.

## 2 Materials and methods

### 2.1 Subjects

We analyzed acute ischemic patients who received EVT in Zhongnan Hospital of Wuhan University from January 2018 to December 2020. Inclusion criteria were as follows: (1) age ≥18 years; (2) conformed to the diagnostic criteria of acute ischemic stroke in Chinese guidelines for diagnosis and treatment of acute ischemic stroke 2018; (3) with LVO in the anterior circulation; (4) received EVT, with or without intravenous alteplase treatment within 4.5 h after symptom onset; and (5) completed 90-day follow-up. Exclusion criteria were as follows: (1) modified Rankin Scale (mRS) score ≥3 before stroke onset; (2) without magnetic resonance imaging or CT examination after EVT; and (3) without complete clinical data. In total, there were 156 patients enrolled in our study. Patients were assigned to the favorable outcome group (90-day mRS ≤2) and the poor outcome group (90-day mRS >2).

Demographic characteristics, including age, gender, mRS score before stroke onset, baseline NIHSS score, blood pressure at admission, baseline blood glucose levels, blood routine test, cholesterol, and kidney function. Cardiovascular risk factors, such as hypertension, diabetes mellitus, hyperlipidemia, atrial fibrillation, history of smoking, and history of ischemic stroke, were recorded as well. Imaging parameters, such as regional leptomeningeal score (rLMC) [25], TAN collateral circulation scores [26], ASPECT score, and hyperdense middle cerebral artery sign, were also analyzed in the study. All images were analyzed by two neuroradiologists with more than 5 years of image analysis experience. Reproducibility of imaging feature quantification was calculated by comparing the results from the two reviewers in 30 random samples.

Successful reperfusion was defined as a modified thrombolysis in cerebral infarction (mTICI) score of 2b or 3. Symptomatic intracerebral hemorrhage (sICH) was defined as any type of ICH with an increase of NIHSS score of ≥4 points compared to baseline within 24 h or leading to death [27]. ASPECT score was divided into dichotomous variables according to <8 and ≥8. TAN scores ≥2 were defined as good collateral circulation, while <2 was defined as poor collateral circulation [26]. Complications included pulmonary infection, gastrointestinal bleeding, and cardiovascular events after EVT.

### 2.2 Primary outcome

The primary outcome was functional dependence (defined as a mRS score of ≤2) at 90 days after EVT [10,11].

### 2.3 Feature selection and model building

First, least absolute shrinkage and selection operator (LASSO) regression algorithm was performed to retain the most predictive features, followed by univariate logistic regression. Variables with a *p* value of <0.05 were incorporated into subsequent models. The importance of the selected variables was evaluated by a random forest algorithm. Then, various machine learning models were built, including logistic regression, LDA, SVM, KNN, and decision tree. Receiver operating characteristic (ROC) curves were constructed, and models were compared by area under the curve (AUC). After comparisons, we chose a multivariate logistic regression algorithm to construct an individualized discriminatory nomogram. Collinearity of combinations of variables in the model was evaluated by variation inflation factors (VIF, <10 indicates no multicollinearity). The concordance index (C-index) and AUC were used to measure the discrimination ability of the nomogram, the calibration plot was performed to visually assess the calibration, and the Hosmer–Lemeshow test was used to evaluate the goodness of fit. Decision curve analysis (DCA), by quantifying net benefits against a range of threshold probabilities, was used to evaluate the clinical utility of the nomogram.

Lastly, a pairwise comparison of ROC curve was used to compare the nomogram with previously published prognostic scoring systems, including THRIVE (Total Health Risks in Vascular Events-calculation) score [12], HAIT2 (Houston Intra-Arterial Therapy 2) score [13], SPAN-100 (Stroke Prognostication using Age and NIHSS) score [14], wSPAN (Weighted Stroke Prognostication using Age and NIHSS) score [15], and PRE (Pittsburgh Response to EVT) score [16]. The THRIVE, HIAT2, SPAN, wSPAN, and PRE index were calculated as previously described [12,13,14,15,16]. The THRIVE score assigned 1 point for age 60–79 years, 2 points for age ≥80 years, 2 points for NIHSS score 11–20, 4 points for NIHSS score ≥21, and 1 point each for hypertension, diabetes mellitus, and atrial fibrillation. The HIAT-2 score assigned 2 points for age 60–79 years, 4 points for age ≥80 years, 1 point for NIHSS score 11–20, 2 points for NIHSS score ≥21, and 3 points for ASPECT score ≤7. The SPAN-100 score was defined as (NIHSS + age), and the score is positive if the sum of age and NIHSS ≥100. wSPAN index was defined as ([3 × NIHSS] + age), and PRE score was defined as (age + 2 × NIHSS − 10 × ASPECT score) score. Furthermore, we made a subgroup analysis of patients in different time windows by the model. The flow of feature selection and model building is shown in Figure S1.

### 2.4 Statistical analysis

Statistical analysis was performed using R software (version 4.1.2, http://www.R-project.org). Continuous variables were expressed as mean ± standard deviation or median (interquartile range, IQR) for normal or non-normal distributions, respectively, followed by an unpaired *t*-test or Wilcoxon rank sum test. Categorical variables were summarized as counts (percentages) and compared using the chi-square test or Fisher’s exact test, as appropriate. All tests with two-sided *p* < 0.05 were considered statistically significant. The R package “tableone” was used in these analyses.

The reproducibility of imaging features of lesion was evaluated by intraclass coefficient (ICC) using a two-way random model with absolute measurements. The “irr” package was used for the ICC algorithm, “tidyverse” package for data collation and exploration, “glmnet” for LASSO regression, “car” for multicollinearity detection, “rms” for nomogram, “Hmisc” for C-index calculation, “rmda” for DCA, “pROC” for ROC analysis, and “ResourceSelection” for goodness of fit test. The packages “MASS,” “e1071,” “class,” “rpart,” “rpart.plot,” “randomForest,” and “caret” were applied in different machine learning models’ construction, respectively.


**Ethical approval:** The research related to human use has been complied with all the relevant national regulations, institutional policies, and in accordance with the tenets of the Helsinki Declaration and has been approved by the authors’ institutional review board or equivalent committee. The studies involving human participants were reviewed and approved by the Ethics Committee of Zhongnan Hospital, Wuhan University (No. 2023007K).
**Informed consent:** Written informed consent for participation was not required for this retrospective study in accordance with the institutional requirement.

## 3 Results

### 3.1 Demographic and clinical characteristics

According to the forementioned inclusion and exclusion criteria, a total of 156 patients were finally enrolled in our study (Table 1). The median age of the patients was 65(55–74) years, and 84 patients (53.8%) were men. An unfavorable outcome at 90 days was observed in 109 (69.9%) of the cases, and the overall mortality at 90 days was 21.2%. The age of patients in the poor outcome group was elder than that in the favorable outcome group (66.00 [57.00, 75.00] vs 62.00 [48.50, 70.00], *p* = 0.04). There was no significant difference in the cardiovascular risk factors and laboratory blood tests between two groups. Compared with the favorable outcome group, the poor outcome group had higher NIHSS score (18.00 [14.00,23.00] vs 13.00 [11.50,15.00], *p* < 0.001), lower rates of ASPECT score ≥8 ( 32 [68.1%] vs 44 [40.4%], *p* = 0.003), successful recanalization (mTICI ≥ 2b) (78 [71.6%] vs 42 [89.4%], *p* = 0.027), and more rates of complications (103 [94.5%] vs 27 [57.4%], *p* < 0.001) and sICH (37 [33.9%] vs 2 [4.3%], *p* < 0.001).

**Table 1:** Demographic and clinical characteristics of patients at baseline

	Overall (*n* = 156)	Favorable outcome (*n* = 47)	Poor outcome (*n* = 109)	*p*
Age (median [IQR])	65.00 [55.00, 74.25]	62.00 [48.50, 70.00]	66.00 [57.00, 75.00]	0.04
Gender, male (*n*, %)	84 (53.8)	22 (46.8)	62 (56.9)	0.326
mRS score before stroke onset (median [IQR])	0.00 [0.00, 0.00]	0.00 [0.00, 0.00]	0.00 [0.00, 0.00]	0.289
Baseline NIHSS score (median [IQR])	17.00 [13.00, 21.00]	13.00 [11.50, 15.00]	18.00 [14.00, 23.00]	<0.001
Previous history				
Hypertension (*n*, %)	82 (52.6)	23 (48.9)	59 (54.1)	0.674
Diabetes (*n*, %)	44 (28.2)	9 (19.1)	35 (32.1)	0.145
Hyperlipidemia (*n*, %)	13 (8.3)	5 (10.6)	8 (7.3)	0.713
Stroke history (*n*, %)	24 (15.4)	3 (6.4)	21 (19.3)	0.071
Atrial fibrillation (*n*, %)	37 (23.7)	10 (21.3)	27 (24.8)	0.791
Coronary heart disease (*n*, %)	26 (16.7)	8 (17.0)	18 (16.5)	1
Smoking (*n*, %)	41 (26.3)	8 (17.0)	33 (30.3)	0.127
SBP (mmHg, mean (SD))	127.61 (23.56)	125.85 (20.90)	128.37 (24.67)	0.542
DBP (mmHg, median [IQR])	73.00 [64.00, 83.25]	72.00 [61.00, 82.00]	74.00 [64.00, 84.00]	0.511
WBC (×10^9^) (median [IQR])	8.15 [6.40, 9.80]	7.97 [6.20, 10.00]	8.20 [6.40, 9.67]	0.818
Neutrophils (×10^9^) (median [IQR])	5.94 [4.66, 7.94]	5.80 [4.72, 7.66]	6.04 [4.68, 8.08]	0.582
Lymphocyte (×10^9^) (median [IQR])	1.15 [0.91, 1.69]	1.27 [0.96, 2.01]	1.14 [0.90, 1.61]	0.219
NLR (median [IQR])	5.02 [3.04, 7.94]	4.97 [2.44, 7.37]	5.14 [3.53, 8.21]	0.232
RBC (×10^12^/L) (median [IQR])	4.23 [3.91, 4.51]	4.30 [3.90, 4.59]	4.21 [3.94, 4.49]	0.443
PLT (×10^9^/L) (mean (SD))	192.11 (57.28)	195.13 (55.35)	190.81 (58.29)	0.667
Hemoglobin (g/L) (median [IQR])	132.30 [124.00, 145.00]	133.00 [120.25, 148.00]	132.00 [125.00, 144.90]	0.842
Cholesterol (mmol/L, median [IQR])	4.32 [3.68, 5.08]	4.42 [4.03, 5.16]	4.27 [3.64, 5.03]	0.295
TG (mmol/L, median [IQR])	1.07 [0.80, 1.58]	1.02 [0.79, 1.94]	1.09 [0.80, 1.56]	0.975
LDL (mmol/L, mean (SD))	2.76 (0.86)	2.59 (0.76)	2.83 (0.89)	0.105
Glucose(mmol/L, median [IQR])	7.56 [6.30, 9.58]	6.90 [6.17, 8.90]	7.80 [6.42, 10.20]	0.028
BUN (mmol/L, median [IQR])	4.72 [4.02, 6.25]	4.69 [4.08, 6.11]	4.73 [3.98, 6.45]	0.885
Scr (μmol/L, median [IQR])	64.25 [53.98, 72.85]	62.20 [52.75, 67.90]	64.80 [55.50, 75.20]	0.214
D-dimer (ng/mL, median [IQR])	447.50 [261.75, 920.00]	356.00 [266.00, 514.50]	501.00 [256.00, 1043.00]	0.181
Thrombolysis before EVT (*n*, %)	74 (47.4)	24 (51.1)	50 (45.9)	0.674
Location of intracranial artery occlusion (*n*, %)				0.689
ICA (*n*, %)	21 (13.5)	8 (17.0)	13 (11.9)	
M1 or M2 segment of MCA (*n*, %)	91 (58.3)	26 (55.3)	65 (59.6)	
Tandem (*n*, %)	44 (28.2)	13 (27.7)	31 (28.4)	
ASPECT score ≥8 (*n*, %)	76 (48.7)	32 (68.1)	44 (40.4)	0.003
HMCAS (*n*, %)	70 (44.9)	17 (36.2)	53 (48.6)	0.208
rMLC (median [IQR])	13.00 [10.00, 15.00]	13.00 [12.00, 15.00]	13.00 [9.00, 15.00]	0.036
Tan score ≥2 (*n*, %)	110 (70.5)	36 (76.6)	74 (67.9)	0.367
Stroke onset to puncture (min, median [IQR])	476.00 [306.00, 705.00]	465.00 [293.00, 647.50]	500.00 [325.00, 705.00]	0.542
Stroke onset to revascularization (min, median [IQR])	601.50 [431.50, 821.50]	526.00 [409.50, 730.50]	626.00 [447.00, 847.00]	0.277
Successful recanalization (*n*, %)	120 (76.9)	42 (89.4)	78 (71.6)	0.027
Complications (*n*, %)	130 (83.3)	27 (57.4)	103 (94.5)	<0.001
Any intracerebral hemorrhage (*n*, %)	90 (57.7)	19 (40.4)	71 (65.1)	0.007
sICH (*n*, %)	39 (25.0)	2 (4.3)	37 (33.9)	<0.001
90-day mRS (median [IQR])	3.00 [2.00, 5.00]	2.00 [1.00, 2.00]	4.00 [3.00, 6.00]	<0.001
Mortality (*n*, %)	33 (21.2)	0(0.0)	33 (30.3)	<0.001

mRS, modified Rankin scale; SBP, systolic blood pressure; DBP, diastolic blood pressure; WBC, white blood cell; RBC, red blood cell; BUN, blood urea nitrogen; NLR, neutrophil-to-lymphocyte ratio; CHOL, total cholesterol; TG, triglyceride; LDL, low-density lipoprotein; ASPECT, Alberta stroke program early CT score; ICA, internal carotid artery; MCA, middle cerebral artery; rLMC, regional leptomeningeal collateral; NIHSS, National Institute of Health Stroke Scale; mTICI, modified thrombolysis in cerebral infarction; HMCAS, hyperdense middle cerebral artery sign.

### 3.2 Identification of significant predictors for poor outcome following EVT

**Figure 1:**
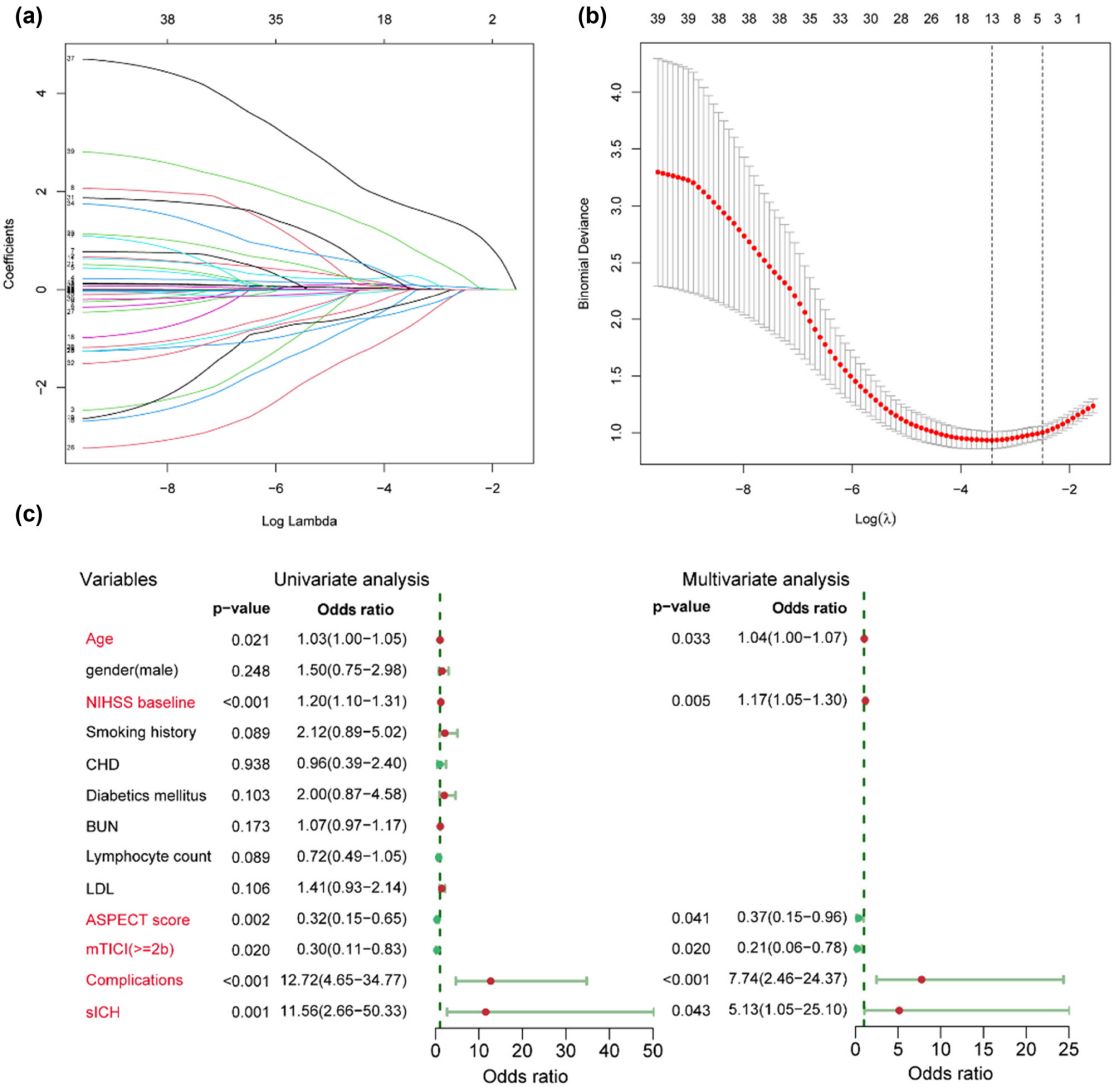
Identification of significant predictors for poor outcome after endovascular treatment in acute anterior circulation stroke patients. (a) LASSO coefficient profiles of the candidate predictors. (b) Selection of the optimal penalization coefficient in LASSO regression. (c) Univariate and multivariate logistic regressions of the predictors.

**Figure 2:**
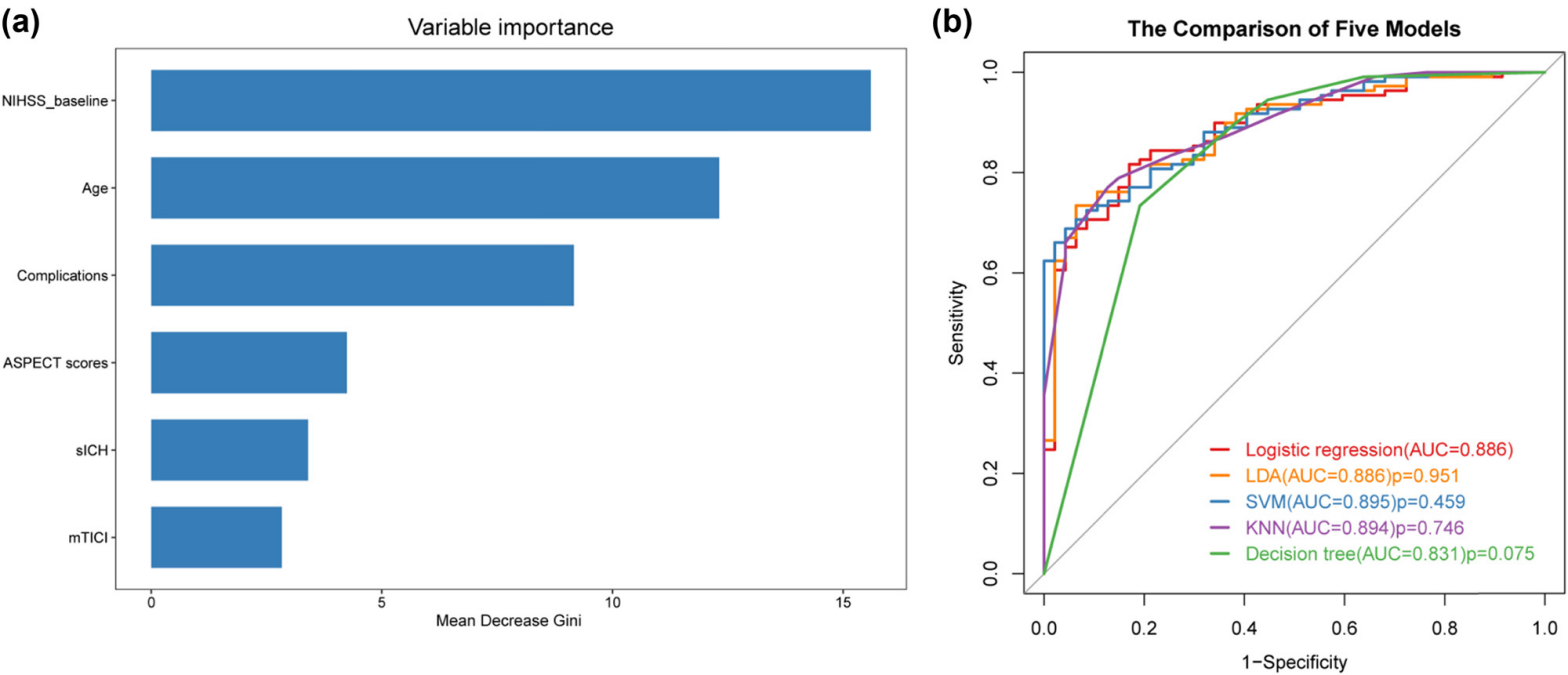
(a) Importance value of clinical variables. Mean Decrease Gini indicates a decrease in the Gini coefficient after variable substitution. A higher value indicates a higher importance. (b) ROC curves of different predictive models using logistic regression (red), LDA (orange), SVM (blue), KNN (purple) method, and decision tree (green).

A total of 39 potential predictors were enrolled in LASSO regression as shown in Figure 1(a) and (b), and 13 candidate predictors with nonzero coefficients were selected, including age, gender, baseline NIHSS score, smoking history, coronary heart disease, diabetic mellitus, blood urea nitrogen, lymphocyte count, low-density lipoprotein, ASPECT score, mTICI score, complications, and sICH. These variables were further analyzed by univariate logistic analysis, and characteristics with *p* < 0.05 were age, baseline NIHSS score, mTICI score, ASPECT score, complications, and sICH (Figure 1c). A variable importance measure was used to evaluate the impact of each variable. The order of importance of the variables was as follows (highest to lowest): baseline NIHSS score, age, complications, ASPECT score, sICH, and mTICI score (Figure 2a). Based on the forementioned significant variables, we constructed different models. The ROC curves indicated that logistic regression was as good as LDA, SVM, and KNN (*p* > 0.05) and non-significantly better than the decision tree (AUC, 0.886 vs 0.831, *p* = 0.075) (Figure 2b, Table 2). Logistic regression model was used for further analysis due to its better performance. No effect of collinearity was observed for the variables in the logistic regression analysis. The results of these variables in the multivariate logistic regression demonstrated that age (odds ratio [OR] = 1.04, 95% confidence interval [CI]: 1.00–1.07, *p* = 0.033), baseline NIHSS score (OR = 1.17, 95% CI: 1.05–1.30, *p* = 0.005), ASPECT score (≥8 vs <8: OR = 0.37, 95% CI: 0.15–0.96, *p* = 0.041), mTICI score (≥2b vs <2b: OR = 0.21, 95% CI: 0.06–0.78, *p* = 0.020), complications (yes vs no: OR = 7.74, 95% CI: 2.46–24.37, *p* < 0.001), sICH (yes vs no: OR = 5.13, 95% CI: 1.05–25.10, *p* = 0.043) were significant independent predictors for outcome after EVT (Figure 1c).

**Table 2:** Differential efficacy of models at optimal predicted probability

Method	Cutoff point	AUC (95% CI)	Sensitivity	Specificity	Positive predictive value	Negative predictive value
Logistic regression	0.695	0.886(0.832–0.940)	0.817	0.830	0.918	0.661
LDA	0.806	0.886(0.834–0.939)	0.734	0.936	0.964	0.603
SVM	0.800	0.895(0.848–0.942)	0.688	0.957	0.974	0.570
KNN	0.731	0.894(0.845–0.942)	0.771	0.872	0.933	0.621
Decision tree	0.782	0.831(0.757–0.905)	0.734	0.809	0.899	0.567

LDA, linear discriminant analysis; SVM, support vector machine; KNN, *k*-nearest neighbors.

### 3.3 Construction and validation of the prediction nomogram

**Figure 3:**
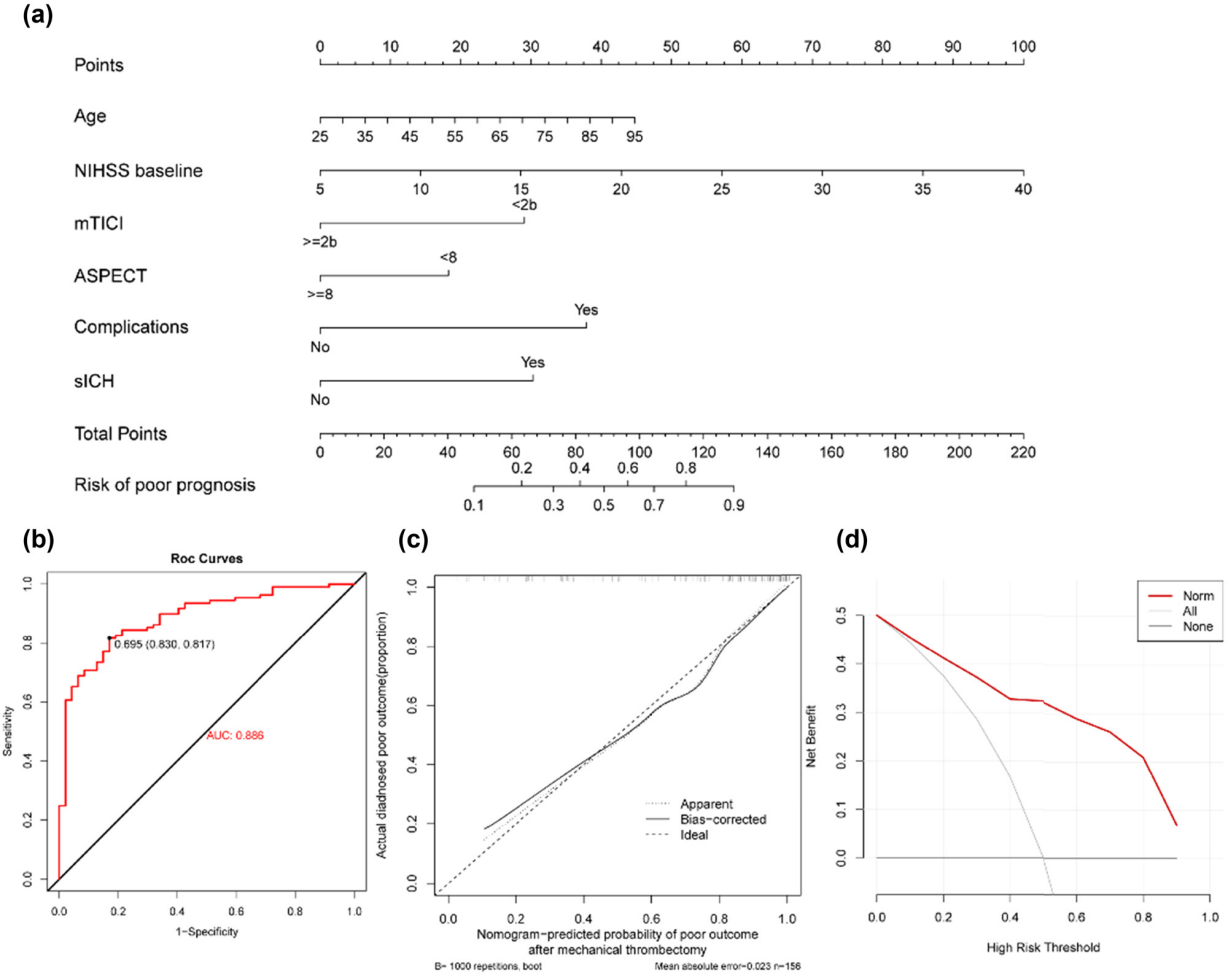
Construction of the predictive nomogram for poor outcome after endovascular treatment in acute anterior circulation stroke patients. (a) Development of the nomogram. For example, if a patient with an age of 55, baseline NIHSS score of 10, mTICI <2b, ASPECT score ≥8, with sICH and without complications, the corresponding points for each factor were 19, 14, 29, 0, 0, and 38, respectively. The total point for this patient was 100, with a probability of 0.65 for having unfavorable outcome after endovascular treatment. (b) ROC curves of the nomogram. (c) Calibration curve of the nomogram. (d) DCA in the cohort.

Based on the above-mentioned significant predicting factors, we established a predictive nomogram. Each of the six predictors was assigned a score ranging from 0 to 100 on a point scale. After adding the scores of all variables to “Total Point”, the corresponding value was the probability of unfavorable outcome event (Figure 3a).

Based on the maximum Youden index, the optimal cutoff value of the nomogram predicted probability was 0.695. At this cutoff value, the sensitivity, specificity, positive predictive value, and negative predictive value for differentiating the presence from the absence of unfavorable outcome events were 81.7, 83.0, 91.8, and 66.1%, respectively. The patient would have poor outcome when the total prediction probability is beyond the cutoff point. ROC curve of the model is demonstrated in Figure 3b. Figure 3c shows the calibration curves for the nomogram model. The calibration curve and an insignificant Hosmer–Lemeshow test statistic (*X*-squared = 7.09, *p* = 0.527) showed a good agreement between the predicted model and actual observation in the study, along with the C-index (0.886, 95% CI 0.832–0.940) indicating that the nomogram is a discriminant tool. Five-fold 100 times cross-validation was performed, in which the C-index was 0.850 (95% CI 0.843–0.857). Given that a C-index >0.75 is generally considered to indicate reliable discrimination, this nomogram performed well in the study. DCA results showed that the nomogram had more benefits in the clinic practice than taking the measures that treat all patients or treat none of patients (Figure 3d).

### 3.4 ROC curve analysis comparing nomogram with other outcome prediction scores

**Figure 4:**
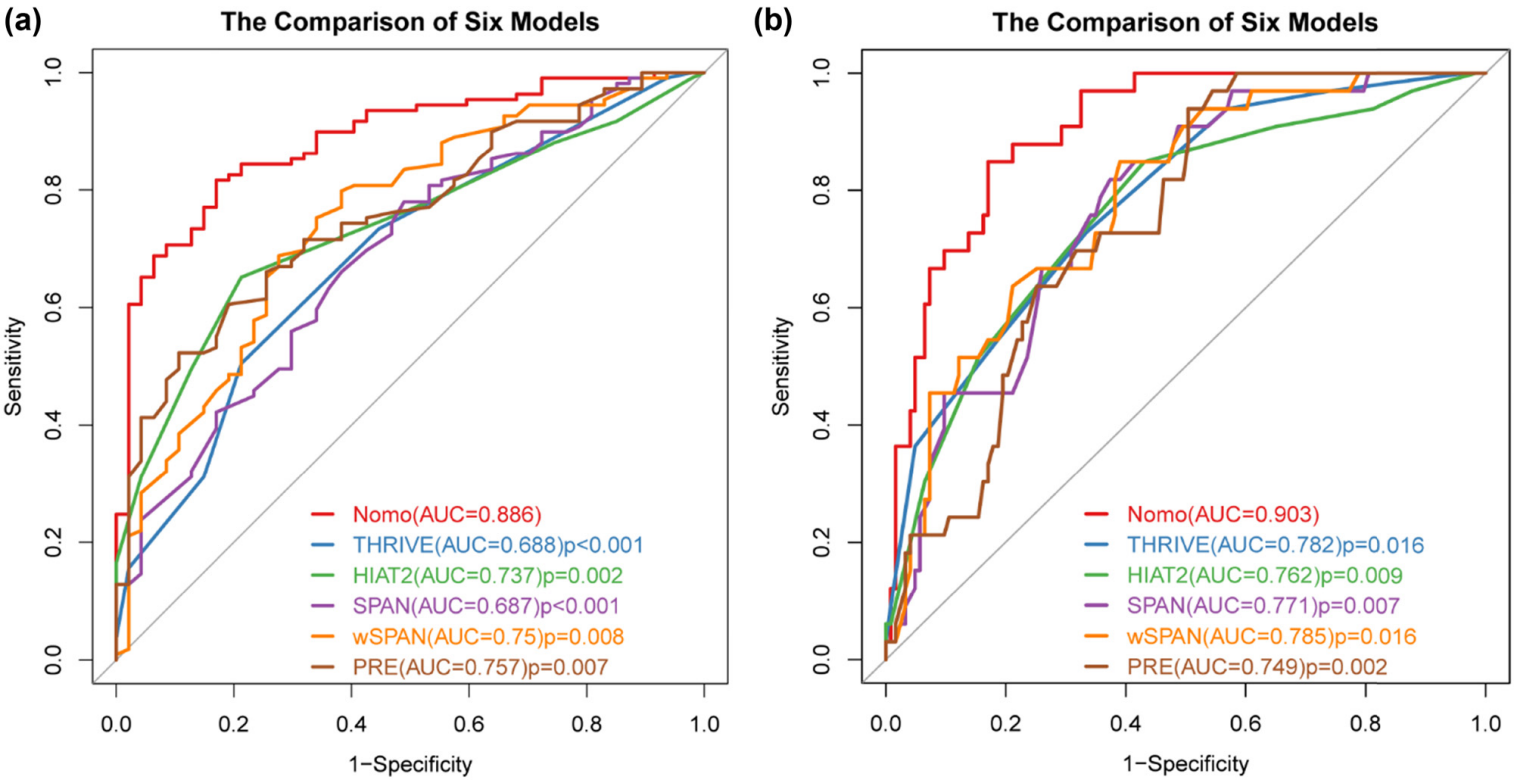
Discriminability analysis. (a) Models predicting poor outcome after EVT. (b) Models predicting mortality after EVT. Comparison of discriminability of the nomogram, THRIVE (Total Health Risks in Vascular Events) score, HAIT2 (Houston Intra-Arterial Therapy 2) score, SPAN-100 (Stroke Prognostication using Age and NIHSS) score, wSPAN (weighted Stroke Prognostication using Age and NIHSS) score, and PRE (Pittsburgh Response to EVT) score was performed by ROC curve analysis. AUC of the nomogram was apparently superior to that of any other prediction model.

We compared the AUC of the nomogram with previously published risk models (Figure 4a). The AUC of the nomogram for predicting poor functional outcome was superior to the THRIVE score (AUC, 0.886 vs 0.688, *p* < 0.001), HAIT2 score (AUC, 0.886 vs 0.737, *p* = 0.002), SPAN-100 score (AUC, 0.886 vs 0.687, *p* < 0.001), wSPAN score (AUC, 0.886 vs 0.750, *p* = 0.008), and PRE score (AUC, 0.886 vs 0.757, *p* = 0.007). This result indicated that our nomogram is a distinctive tool for the outcome prediction after EVT. A similar significant difference was also observed in the prediction of mortality (Figure 4b). Besides, our nomogram was superior to the other two prediction models [28,29], as it is shown in Figure S2.

### 3.5 Subgroup analysis in patients with different time windows

We divided patients into two groups according to whether they were admitted to hospital within 6 h. The AUC of the nomogram in the within 6-h time window group was 0.914 (95% CI, 0.833–0.995), and the AUC in the other group was 0.889 (95% CI, 0.828–0951).

### 3.6 Reproducibility of imaging data

The ICCs of the imaging data for the two reviewers in measuring mTICI, infarct site, ASPECT score, hyperdense middle cerebral artery sign, rMLC, and TAN score were 0.819, 0.929, 0.963, 0.806, 0.747, and 0.804, respectively.

## 4 Discussion

It is of great clinical value to predict the functional outcome of acute ischemic stroke patients. In this study, we established and compared various machine learning models to predict functional outcomes after EVT for acute ischemic stroke due to LVO in the anterior circulation. We screened variables by Lasso regression and univariate logistic regression, demonstrating that prognostic factors were age, admission NIHSS score, mTICI score, sICH, baseline ASPECT score, and complications. The combination of these variables was compared in different machine learning methods. Furthermore, a reliable nomogram model based on these selected variables was developed to predict the probability of poor outcome following EVT.

Previous studies mainly analyzed patients who received EVT within 6 h after stroke onset and may not be suitable for patients with longer time window. Studies have confirmed that patients with stroke onset within 6–24 h who received CT perfusion evaluation and met the DAWN or DEFUSE 3 eligibility criteria could benefit from the treatment of EVT [8,9]. In addition, a recent meta-analysis strengthened the evidence of benefit of EVT in patients with sufficient reversible cerebral ischemia within the time window of 6–24 h [30]. The result showed that EVT was associated with a higher rate of functional independence than best medical therapy alone (the proportion of mRS 0–2, 45.9% vs 19.3%; *p* < 0.001). In our study, we included patients who underwent EVT for acute anterior circulation LVO stroke patients within 24 h, constructed a nomogram, and performed a subgroup analysis, showing that our model has good predictive ability.

Up to now, several prognostic assessment systems have been applied to patients after EVT. However, their applicability for predicting 3-month functional outcome showed a moderate discriminative performance. The THRIVE score included age, admission NIHSS score, and medical history, but without laboratory data and imaging features [12]. The predictive accuracy of the HIAT2 score was limited by categorizing the continuous variables including age, baseline NIHSS score, blood glucose, and ASPECT score [13]. Although the wSPAN score and the PRE score obtained a higher predictive accuracy than the SPAN-100 score when applied in the present study population (AUC were 0.750, 0.757, and 0.687, respectively), all of them presented unsatisfactory predictive efficacy. The novel nomogram in our study demonstrated a good predictive power with good discrimination (C-index 0.886, 95% CI 0.832–0.940) and calibration (Hosmer–Lemeshow test, *X*-squared = 7.09, *p* = 0.527). The result of the internal cross-validation by bootstrap analysis further confirmed the reliability of the nomogram. Besides, DCA further showed that our nomogram conferred significantly high clinical net benefits. Collectively, our nomogram presented a potential value for individual assessment of functional outcome in anterior circulation stroke patients following EVT; therefore, it could provide information for early identification of patients who are appropriate candidates for post-EVT intensive management.

Consistent with most previous prediction models, baseline NIHSS score and age were the most predictive factors for 3-month outcome after EVT [12,13,14,15,16]. According to our variable importance analysis, NIHSS score and age ranked first and second in importance, and the result was basically in line with the findings of Ospel et al. [15], which showed that a 1-point increase in NIHSS score roughly corresponded to a 3-year increase in patient’s age. NHISS score represents the severity of nerve damage. The higher the NIHSS is, the greater the possibility of unfavorable prognosis after EVT will be. The increase in age means the decline in organ function, not only the reduction of intracranial collateral circulation and poor reperfusion but also the decline of immunity, and the increase of postoperative infection and other complications. Several studies have demonstrated that despite successful revascularization, increasing age and high NIHSS score were still associated with unfavorable outcome [31,32]. These data indicate that it is a great challenge for EVT to reverse severe neurological dysfunction in elderly patients.

The ASPECT score was used to assess early ischemic changes on computed tomography [33]. It has been universally applied since the pre-thrombectomy era as a pragmatic, reliable, and easily applicable scoring template and was used for patient selection in clinical trials [34]. Another major focus of thrombectomy is successful recanalization. Rapid, early, and effective recanalization to rescue the ischemic penumbra is a strong predictor of good prognosis in random control trials [2,3,4,5,6,35]. Consistent with previous studies [7,36,37,38], lower ASPECT score (≤7) and unsuccessful recanalization (mTICI 0-2a) predict an unfavorable outcome in patients after EVT in our model.

In majority of previous studies, sICH was considered a secondary outcome after EVT, indicating its importance in the overall prognosis [36,39]. sICH could predict further neurological impairment, secondary cerebral edema, intracranial hypertension, and even death [10,11,40,41]. Few studies have investigated complications of other organs in stroke patients after EVT, even though poor recovery and high mortality rate after EVT are largely due to several complications, such as pneumonia, severe infection, gastrointestinal hemorrhage, and cardiovascular events. Stroke patients with LVO usually present severe symptoms, low consciousness, throat paralysis, and prolonged bedridden, increasing the risk of infection [42]. In addition, gastrointestinal bleeding caused by stress ulcer after EVT has always been a problem for clinicians, which limits the use of antiplatelet agents and increases the risk of arterial re-occlusion, leading to catastrophic consequences [43]. Cardiovascular events, such as myocardial infarction and heart failure, can also affect the prognosis of stroke patients, leading to stroke recurrence and death [44]. To sum up, these complications, as an independent risk factor in our model, should be considered seriously in the management of post-procedure besides sICH.

Previous evidence suggested that the extent of hemodynamic impairment on CT perfusion before treatment had limited predictive value after EVT [45]. Our results with available data presented a similar opinion. As shown in Figure S3 and Table S1, compared to the poor outcome group, patients with favorable prognosis had smaller infarct core, larger ischemic penumbra, and larger mismatch volume; however, there was no statistical significance between the two groups. It should be viewed with caution due to the small sample size in the present study.

Several studies have confirmed collateral circulation plays a vital role in maintaining the blood supply to brain tissue and vessels within the oligemic regions. Hence, poor collateral circulation would aggravate blood vessel damage, leading to reperfusion injury and hemorrhagic transformation after revascularization therapy [46,47]. In our study, the collateral circulation score (TAN score, rLMC score) failed to finalize into the model, and this may be related to variable screening in the LASSO algorithm, which implies that other variables (such as sICH, complications) are better predictors of poor prognosis after EVT than collateral circulation scores. However, this requires more studies to flesh out our results.

Hyperdense middle cerebral artery sign (HMCAS) on non-contrast head CT scan of AIS patients, reflecting the presence of an intravascular thrombus rich in red blood cells in middle cerebral artery territory, has demonstrated that it increases the risk of poor outcomes in AIS patients treated with IV thrombolysis in several studies [48,49]. In our study, patients with HMCAS approximately account for 44.9%. Comparing patients in favorable outcome groups, those in poor prognosis groups have more frequency in HMCAS (36.2 vs 48.6%); however, statistical comparisons did not reveal meaning result (*p* = 0.208). The finding shows that in patients due to LVO treated with EVT, those with HMCAS might not have a worse outcome at 3 months compared to those with no-HMCAS, and it is basically in line with a recently published study [50].

Several limitations of our study should be noted. First, the nature of retrospective single-center study with a small sample size limited the generalization of the conclusion to other geographical and ethnic groups. A prospective multicenter study is required to confirm the validity of this model. Second, some potential variables were not included in our study, such as final infarct volume and arterial re-occlusion rate after EVT. Nevertheless, we confirmed the effectiveness of our model via comparing to different machine learning algorithms and enhanced the reliability of the model.

In conclusion, our nomogram composed of age, NIHSS score at admission, baseline ASPECT score, recanalization status, sICH, and complications could predict 3-month outcome in LVO-ischemic stroke patients treated with EVT. The discrimination and calibration of the nomogram performed well in internal validation. The AUC of the nomogram was higher than that of several previous prediction assessment systems. Further studies in other populations and areas are warranted to validate the efficacy of the nomogram.

## Supplementary Material

Supplementary material

## References

[1] Campbell BCV, Silva DAD, Macleod MR, Coutts SB, Schwamm LH, Davis SM, et al. Ischaemic stroke. Nat Rev Dis Primers. 2019;5(1):1–22. 10.1038/s41572-019-0118-8 31601801

[2] Saver JL, Goyal M, Bonafe A, Diener HC, Levy EI, Pereira VM, et al. Stent-retriever thrombectomy after intravenous t-PA vs t-PA alone in stroke. N Engl J Med. 2015;372(24):2285–95. 10.1056/NEJMoa1415061 25882376

[3] Berkhemer OA, Fransen PSS, Beumer D, van den Berg LA, Lingsma HF, Yoo AJ, et al. A randomized trial of intraarterial treatment for acute ischemic stroke. N Engl J Med. 2015;372(1):11–20. 10.1056/NEJMoa1411587 25517348

[4] Campbell BCV, Mitchell PJ, Kleinig TJ, Dewey HM, Churilov L, Yassi N, et al. Endovascular therapy for ischemic stroke with perfusion-imaging selection. N Engl J Med. 2015;372(11):1009–18. 10.1056/NEJMoa1414792 25671797

[5] Goyal M, Demchuk AM, Menon BK, Eesa M, Rempel JL, Thornton J, et al. Randomized assessment of rapid endovascular treatment of ischemic stroke. N Engl J Med. 2015;372(11):1019–30. 10.1056/NEJMoa1414905 25671798

[6] Jovin TG, Chamorro A, Cobo E, de Miquel MA, Molina CA, Rovira A, et al. Thrombectomy within 8 hours after symptom onset in ischemic stroke. N Engl J Med. 2015;372(24):2296–306. 10.1056/NEJMoa1503780 25882510

[7] Goyal M, Menon BK, van Zwam WH, Dippel DWJ, Mitchell PJ, Demchuk AM, et al. Endovascular thrombectomy after large-vessel ischaemic stroke: a meta-analysis of individual patient data from five randomised trials. Lancet. 2016;387(10029):1723–31. 10.1016/S0140-6736(16)00163-X 26898852

[8] Nogueira RG, Jadhav AP, Haussen DC, Bonafe A, Budzik RF, Bhuva P, et al. Thrombectomy 6–24 hours after stroke with a mismatch between deficit and infarct. N Engl J Med. 2018;378(1):11–21. 10.1056/NEJMoa1706442 29129157

[9] Albers GW, Marks MP, Kemp S, Christensen S, Tsai JP, Ortega-Gutierrez S, et al. Thrombectomy for stroke at 6 to 16 hours with selection by perfusion imaging. N Engl J Med. 2018;378(8):708–18. 10.1056/NEJMoa1713973 PMC6590673 29364767

[10] LeCouffe NE, Kappelhof M, Treurniet KM, Rinkel LA, Bruggeman AE, Berkhemer OA, et al. A randomized trial of intravenous alteplase before endovascular treatment for stroke. N Engl J Med. 2021;385(20):1833–44. 10.1056/NEJMoa2107727 34758251

[11] Yang P, Zhang Y, Zhang L, Zhang Y, Treurniet KM, Chen W, et al. Endovascular thrombectomy with or without intravenous alteplase in acute stroke. N Engl J Med. 2020;382(21):1981–93. 10.1056/NEJMoa2001123 32374959

[12] Flint AC, Xiang B, Gupta R, Nogueira RG, Lutsep HL, Jovin TG, et al. THRIVE score predicts outcomes with a third-generation endovascular stroke treatment device in the TREVO-2 trial. Stroke. 2013;44(12):3370–5. 10.1161/STROKEAHA.113.002796 PMC4157913 24072003

[13] Sarraj A, Albright K, Barreto AD, Boehme AK, Sitton CW, Choi J, et al. Optimizing prediction scores for poor outcome after intra-arterial therapy in anterior circulation acute ischemic stroke. Stroke. 2013;44(12):3324–30. 10.1161/STROKEAHA.113.001050 PMC4135710 23929748

[14] Saposnik G, Guzik AK, Reeves M, Ovbiagele B, Johnston SC. Stroke prognostication using age and NIH stroke scale: SPAN-100. Neurology. 2013;80(1):21–8. 10.1212/WNL.0b013e31827b1ace PMC3589202 23175723

[15] Ospel JM, Brown S, Kappelhof M, van Zwam W, Jovin T, Roy D, et al. Comparing the prognostic impact of age and baseline national institutes of health stroke scale in acute stroke due to large vessel occlusion. Stroke. 2021;52(9):2839–45. 10.1161/STROKEAHA.120.032364 34233465

[16] Ali Raza S, Xiang B, Jovin TG, Liebeskind DS, Shields R, Nogueira RG, et al. Pittsburgh response to endovascular therapy score as a pre-treatment prognostic tool: External validation in Trevo2. Int J Stroke. 2017;12(5):494–501. 10.1177/1747493016677984 27811307

[17] Cheng Z, Geng X, Rajah GB, Gao J, Ma L, Li F, et al. NIHSS consciousness score combined with ASPECTS is a favorable predictor of functional outcome post endovascular recanalization in stroke patients. Aging Dis. 2021;12(2):415–24. 10.14336/AD.2020.0709 PMC7990364 33815874

[18] Cheng WC, Chen LH, Jiang CR, Deng YM, Wang DW, Lin CH, et al. Sensible functional linear discriminant analysis effectively discriminates enhanced raman spectra of mycobacterium species. Anal Chem. 2021;93(5):2785–92. 10.1021/acs.analchem.0c03681 33480698

[19] Huang S, Cai N, Pacheco PP, Narrandes S, Wang Y, Xu W. Applications of support vector machine (SVM) learning in cancer genomics. Cancer Genomics Proteom. 2018;15(1):41–51. 10.21873/cgp.20063 PMC5822181 29275361

[20] Saçlı B, Aydınalp C, Cansız G, Joof S, Yilmaz T, Çayören M, et al. Microwave dielectric property based classification of renal calculi: Application of a kNN algorithm. Comput Biol Med. 2019;112:103366. 10.1016/j.compbiomed.2019.103366 31386972

[21] Flayer CH, Perner C, Sokol CL. A decision tree model for neuroimmune guidance of allergic immunity. Immunol Cell Biol. 2021;99(9):936–48. 10.1111/imcb.12486 PMC8490305 34115905

[22] Iasonos A, Schrag D, Raj GV, Panageas KS. How to build and interpret a nomogram for cancer prognosis. J Clin Oncol. 2008;26(8):1364–70. 10.1200/JCO.2007.12.9791 18323559

[23] Hijazi Z, Oldgren J, Lindbäck J, Alexander JH, Connolly SJ, Eikelboom JW, et al. The novel biomarker-based ABC (age, biomarkers, clinical history)-bleeding risk score for patients with atrial fibrillation: a derivation and validation study. Lancet. 2016;387(10035):2302–11. 10.1016/S0140-6736(16)00741-8 27056738

[24] Liu Z, Zhong F, Xie Y, Lu X, Hou B, Ouyang K, et al. A predictive model for the risk of posterior circulation stroke in patients with intracranial atherosclerosis based on high resolution MRI. Diagnostics (Basel). 2022;12(4):812. 10.3390/diagnostics12040812 PMC9031625 35453860

[25] Menon BK, Smith EE, Modi J, Patel SK, Bhatia R, Watson TWJ, et al. Regional leptomeningeal score on CT angiography predicts clinical and imaging outcomes in patients with acute anterior circulation occlusions. AJNR Am J Neuroradiol. 2011;32(9):1640–5. 10.3174/ajnr.A2564 PMC7965388 21799045

[26] Tan IYL, Demchuk AM, Hopyan J, Zhang L, Gladstone D, Wong K, et al. CT angiography clot burden score and collateral score: correlation with clinical and radiologic outcomes in acute middle cerebral artery infarct. AJNR Am J Neuroradiol. 2009;30(3):525–31. 10.3174/ajnr.A1408 PMC7051470 19147716

[27] Hacke W, Kaste M, Fieschi C, von Kummer R, Davalos A, Meier D, et al. Randomised double-blind placebo-controlled trial of thrombolytic therapy with intravenous alteplase in acute ischaemic stroke (ECASS II). Second European-Australasian Acute Stroke Study Investigators. Lancet. 1998;352(9136):1245–51. 10.1016/s0140-6736(98)08020-9 9788453

[28] Sun H, Zhou F, Zhang G, Hou J, Liu Y, Chen X, et al. A novel nomogram for predicting prognosis after mechanical thrombectomy in patients with acute ischemic stroke. Curr Neurovasc Res. 2021;18(5):479–88. 10.2174/1567202618666211210154739 34895124

[29] Meng L, Wang H, Yang H, Zhang X, Zhang Q, Dong Q, et al. Nomogram to predict poor outcome after mechanical thrombectomy at older age and histological analysis of thrombus composition. Oxid Med Cell Longev. 2020;2020:8823283. 10.1155/2020/8823283 PMC7765717 33381271

[30] Jovin TG, Nogueira RG, Lansberg MG, Demchuk AM, Martins SO, Mocco J, et al. Thrombectomy for anterior circulation stroke beyond 6 h from time last known well (AURORA): a systematic review and individual patient data meta-analysis. Lancet. 2022;399(10321):249–58. 10.1016/S0140-6736(21)01341-6 34774198

[31] Shi ZS, Liebeskind DS, Xiang B, Ge SG, Feng L, Albers GW, et al. Predictors of functional dependence despite successful revascularization in large-vessel occlusion strokes. Stroke. 2014;45(7):1977–84. 10.1161/STROKEAHA.114.005603 PMC4160889 24876082

[32] van Horn N, Kniep H, Leischner H, McDonough R, Deb-Chatterji M, Broocks G, et al. Predictors of poor clinical outcome despite complete reperfusion in acute ischemic stroke patients. J Neurointerv Surg. 2021;13(1):14–8. 10.1136/neurintsurg-2020-015889 32414889

[33] Barber PA, Demchuk AM, Zhang J, Buchan AM. Validity and reliability of a quantitative computed tomography score in predicting outcome of hyperacute stroke before thrombolytic therapy. ASPECTS Study Group. Alberta Stroke Programme Early CT Score. Lancet. 2000;355(9216):1670–4. 10.1016/s0140-6736(00)02237-6 10905241

[34] Nezu T, Koga M, Kimura K, Shiokawa Y, Nakagawara J, Furui E, et al. Pretreatment ASPECTS on DWI predicts 3-month outcome following rt-PA: SAMURAI rt-PA Registry. Neurology. 2010;75(6):555–61. 10.1212/WNL.0b013e3181eccf78 20697108

[35] Loo JH, Leow AS, Jing M, Sia CH, Chan BP, Seet RC, et al. Impact of atrial fibrillation on the treatment effect of bridging thrombolysis in ischemic stroke patients undergoing endovascular thrombectomy: a multicenter international cohort study. J Neurointerv Surg. 2023 Dec;15(12):1274–9. 10.1136/jnis-2022-019590 36609541

[36] Chen Y, Zhou S, Yang S, Mofatteh M, Hu Y, Wei H, et al. Developing and predicting of early mortality after endovascular thrombectomy in patients with acute ischemic stroke. Front Neurosci. 2022 Dec;16:1034472. 10.3389/fnins.2022.1034472. PMC9810273 36605548

[37] Lai Y, Diana F, Mofatteh M, Nguyen TN, Jou E, Zhou S, et al. Predictors of failure of early neurological improvement in early time window following endovascular thrombectomy: a multi-center study. Front Neurol. 2023 Sep;14:1227825. 10.3389/fneur.2023.1227825 PMC10538528 37780716

[38] Yoo AJ, Zaidat OO, Chaudhry ZA, Berkhemer OA, González RG, Goyal M, et al. Impact of pretreatment noncontrast CT Alberta Stroke Program Early CT Score on clinical outcome after intra-arterial stroke therapy. Stroke. 2014;45(3):746–51. 10.1161/STROKEAHA.113.004260 24503670

[39] Chen Y, Nguyen TN, Mofatteh M, Abdalkader M, Wellington J, Yan Z, et al. Association of early increase in body temperature with symptomatic intracranial hemorrhage and unfavorable outcome following endovascular therapy in patients with large vessel occlusion stroke. J Integr Neurosci. 2022 Sep;21(6):156. 10.31083/j.jin2106156 36424759

[40] Zi W, Qiu Z, Li F, Sang H, Wu D, Luo W, et al. Effect of endovascular treatment alone vs intravenous alteplase plus endovascular treatment on functional independence in patients with acute ischemic stroke: The DEVT randomized clinical trial. JAMA. 2021;325(3):234–43. 10.1001/jama.2020.23523 PMC7816099 33464335

[41] Suzuki K, Matsumaru Y, Takeuchi M, Morimoto M, Kanazawa R, Takayama Y, et al. Effect of mechanical thrombectomy without vs with intravenous thrombolysis on functional outcome among patients with acute ischemic stroke: The SKIP randomized clinical trial. JAMA. 2021;325(3):244–53. 10.1001/jama.2020.23522 PMC7816103 33464334

[42] Maier IL, Schramm K, Bähr M, Behme D, Psychogios MN, Liman J. Predictive factors for the need of tracheostomy in patients with large vessel occlusion stroke being treated with mechanical thrombectomy. Front Neurol. 2021;12:728624. 10.3389/fneur.2021.728624 PMC8660673 34899559

[43] Du W, Zhao X, Wang Y, Pan Y, Liu G, Wang A, et al. Gastrointestinal bleeding during acute ischaemic stroke hospitalisation increases the risk of stroke recurrence. Stroke Vasc Neurol. 2020;5(2):116–20. 10.1136/svn-2019-000314 PMC7337367 32606083

[44] Böhm M, Schumacher H, Teo KK, Lonn EM, Lauder L, Mancia G, et al. Cardiovascular outcomes in patients at high cardiovascular risk with previous myocardial infarction or stroke. J Hypertens. 2021;39(8):1602–10. 10.1097/HJH.0000000000002822 34188004

[45] Brugnara G, Neuberger U, Mahmutoglu MA, Foltyn M, Herweh C, Nagel S, et al. Multimodal predictive modeling of endovascular treatment outcome for acute ischemic stroke using machine-learning. Stroke. 2020;51(12):3541–51. 10.1161/STROKEAHA.120.030287 33040701

[46] Chalos V, Venema E, Mulder MJHL, Roozenbeek B, Steyerberg EW, Wermer MJH, et al. Development and validation of a postprocedural model to predict outcome after endovascular treatment for ischemic stroke. JAMA Neurol. 2023 Jul;80(9):940–8. 10.1001/jamaneurol.2023.2392 PMC10391355 37523199

[47] Cao R, Lu Y, Qi P, Wang Y, Hu H, Jiang Y, et al. Collateral circulation and BNP in predicting outcome of acute ischemic stroke patients with atherosclerotic versus cardioembolic cerebral large-vessel occlusion who underwent endovascular treatment. Brain Sci. 2023 Mar;13(4):539. 10.3390/brainsci13040539 PMC10137090 37190504

[48] Paliwal PR, Ahmad A, Shen L, Yeo LL, Loh PK, Ng KW, et al. Persistence of hyperdense middle cerebral artery sign on follow-up CT scan after intravenous thrombolysis is associated with poor outcome. Cerebrovasc Dis. 2012;33(5):446–52. 10.1159/000336863 22456065

[49] Sun H, Liu Y, Gong P, Zhang S, Zhou F, Zhou J. Intravenous thrombolysis for ischemic stroke with hyperdense middle cerebral artery sign: A meta-analysis. Acta Neurol Scand. 2020 Mar;141(3):193–201. 10.1111/ane.13177 31598961

[50] Chen Y, Diana F, Mofatteh M, Zhou S, Chen J, Huang Z, et al. Functional and technical outcomes in acute ischemic stroke patients with hyperdense middle cerebral artery sign treated with endovascular thrombectomy. Front Neurol. 2023 May;14:1150058. 10.3389/fneur.2023.1150058 PMC10247996 37305752

