# Supplementary material for: Predicting functional outcome in acute ischemic stroke patients after endovascular treatment by machine learning
Source: Transl Neurosci. 2023 Nov 27;14(1):20220324. doi: 10.1515/tnsci-2022-0324 (PMC10685342; doi:10.1515/tnsci-2022-0324)
Supplement: Supplementary material [file tnsci-2022-0324-sm.pdf]

Supplementary material

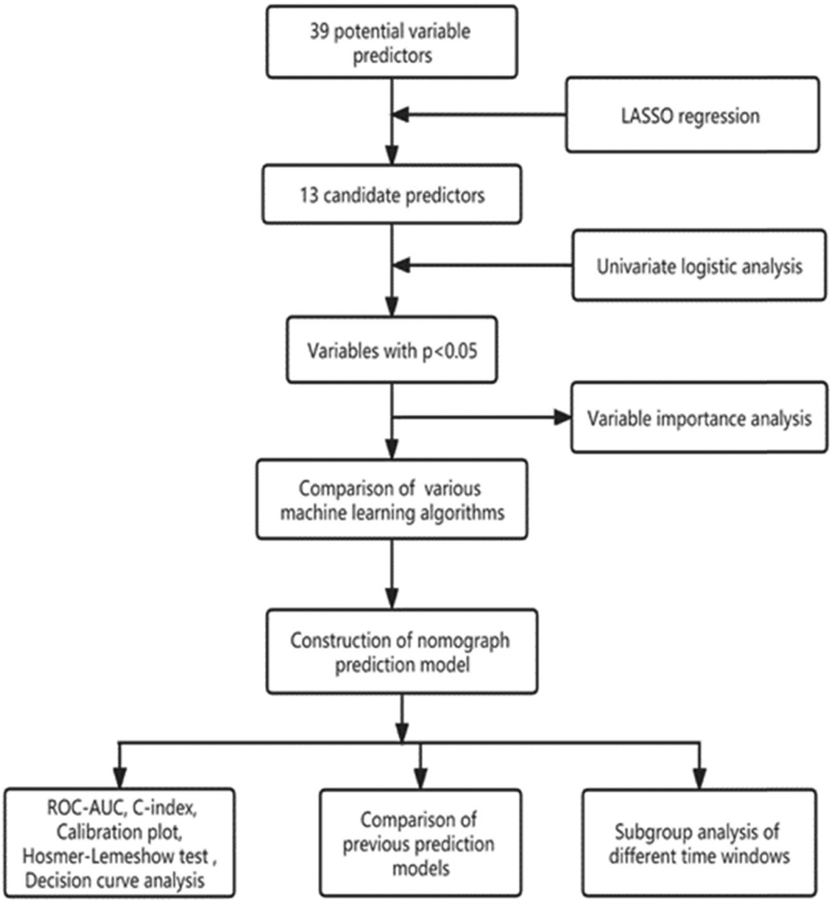

Figure S1: The flow chart of analysis process.

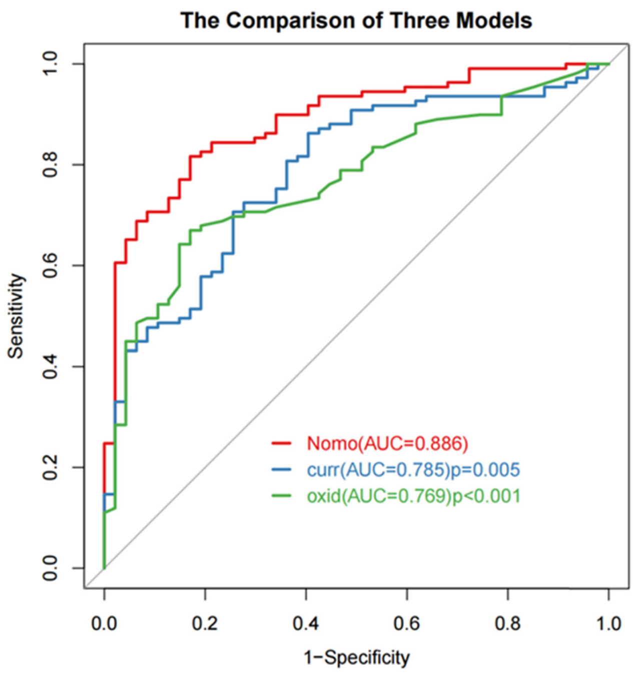

Figure S2: Discriminability analysis in three models. curr: PMID: 34895124, oxid: PMID: 33381271.

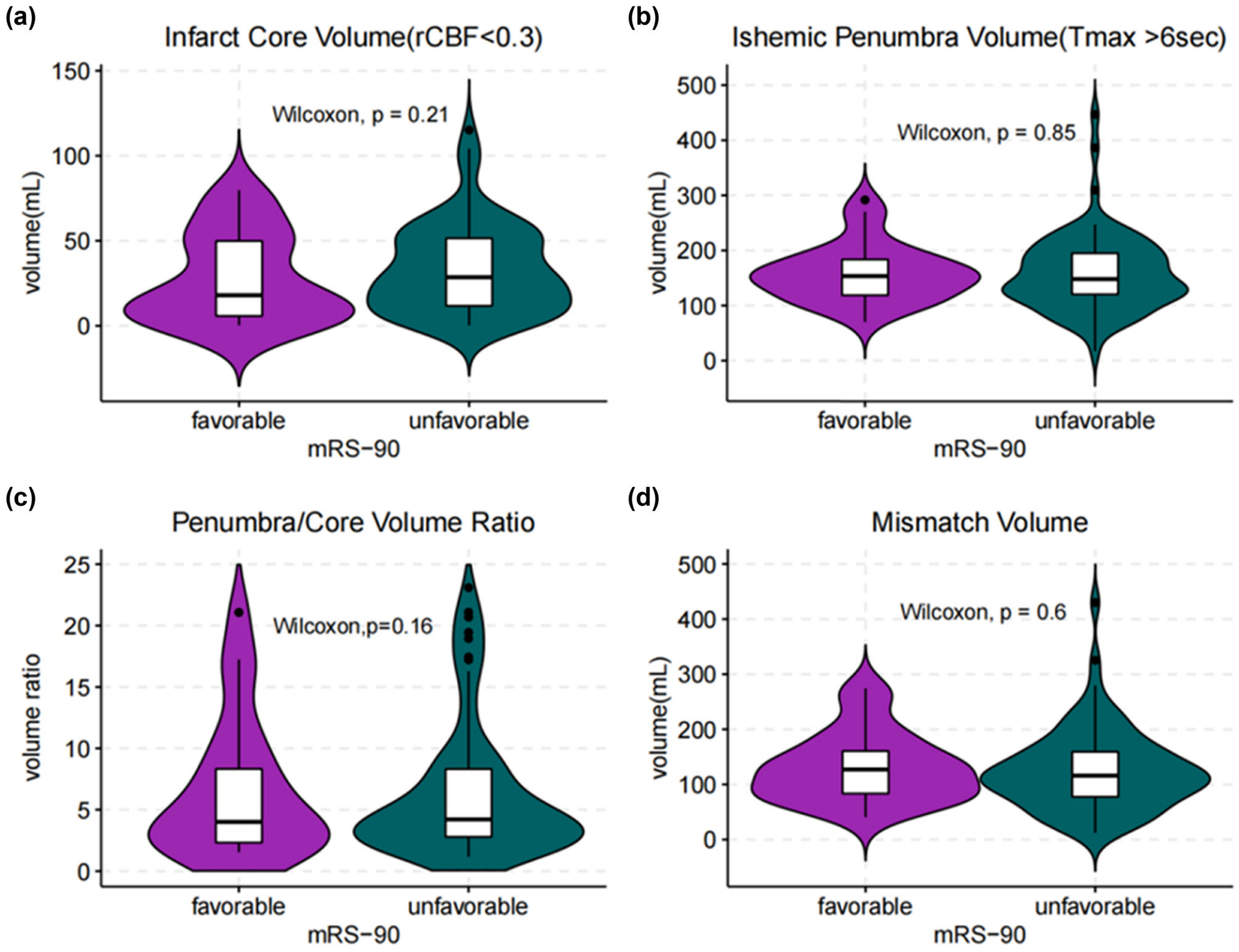

Figure S3: Comparison of computed tomography perfusion (CTP) parameters (A. infarct core volume, B. ischemic penumbra volume, C. penumbra/core volume ratio, D. mismatch volume) at baseline between patients in the favorable and unfavorable outcome group.

Table S1: Comparison of ischemic core and ischemic penumbra volume between favorable and unfavorable outcome groups

	**Overall (*n* = 105)**	**Favorable outcome (*n* = 30)**	**Unfavorable outcome (*n* = 75)**	***p***
Ischemic core (rCBF <30%) (ml, median [IQR])	24.83 [8.50, 51.29]	18.00 [5.82, 49.85]	28.60 [11.76, 51.44]	0.213
Ischemic penumbra volume (ml, median [IQR])	151.40 [119.70, 191.40]	153.22 [118.56, 183.32]	147.80 [120.15, 194.57]	0.843
Mismatch volume (ml, median [IQR])	118.02 [79.87, 160.50]	127.16 [83.56, 160.62]	116.03 [77.62, 159.25]	0.595
Penumbra/core volume ratio (median [IQR])	5.41 [2.86, 17.25]	8.10 [2.79, 43.02]	4.96 [2.91, 13.23]	0.162
